# Supplementary material for: Optimization of Sample Preparation for Detection of 10 Phthalates in Non-Alcoholic Beverages in Northern Vietnam
Source: Toxics. 2018 Nov 19;6(4):69. doi: 10.3390/toxics6040069 (PMC6316763; doi:10.3390/toxics6040069)
Supplement: Supplementary file 1 [file toxics-06-00069-s001.pdf]

Supplementary Materials: Optimization of Sample Preparation for Detection of 10 Phthalates in Non-Alcoholic Beverages in Northern Vietnam

Thanh-Thien Tran-Lam, Yen Hai Dao, Duong Thanh Nguyen, Hoi Kim Ma, Trung Quoc Pham and Giang Truong Le

Table S1. Identifying, quantifying ions, retention time, and collision energy of 13 phthalates.

Abbr.	Compound	t_R (min)	Prec. (*m/z*)	Frag. (*m/z*)	CE (eV)	Remark
DMP	Dimethyl phthalate	6.58	163.0	77.1 (51.1)	20 (14)	Q (C)
DEP	Diethyl phthalate	8.16	177.0	149.0 (65.1)	8 (20)	Q (C)
DPP	Dipropyl phthalate	10.12	149.0	65.1 (121.0)	20 (12)	Q (C)
DiBP	Diisobutyl phthalate	12.00	149.0	65.1 (121.0)	22 (12)	Q (C)
BzDP	Benzyl butyl phthalate	15.52	206.1	149.1 (93.0)	8 (16)	Q (C)
DnHP	Di-n-hexyl phthalate	15.42	149.0	65.0 (93.0)	22 (16)	Q (C)
DEHP	Di(2-ethylhexyl) phthalate	17.02	167.1	149.0 (65.1)	6 (22)	Q (C)
DnOP	Di-n-octyl phthalate	18.42	150.1	66.1 (93.0)	22 (16)	Q (C)
DCHP	Dicyclohexyl phthalate	16.90	167.1	149.1 (105.1)	8 (14)	Q (C)
DBP	Di-n-butyl phthalate	12.33	149.0	65.1 (93.0)	20 (12)	Q (C)
DMP-d4	Dimethyl phthalate-3,4,5,6-d4	6.58	167.1	81.1 (139.1)	20 (10)	Q (C)
DiBP-d4	Diisobutyl phthalate-3,4,5,6-d4	12.00	153.0	69.1 (97.1)	20 (15)	Q (C)
DEHP-d4	Di(2-ethylhexyl) phthalate-3,4,5,6-d4	17.02	171.1	153.0 (125.1)	15 (20)	Q (C)

Note: The value in parenthesis represent Confirm (C). Retention time (t_R); Fragment (m/z), Quantitation (Q); Confirm (C); and Collision energy (eV).
